# The oncoprotein gankyrin promotes the development of colitis-associated cancer through activation of STAT3

**DOI:** 10.18632/oncotarget.14983

**Published:** 2017-02-01

**Authors:** Toshiharu Sakurai, Hiroaki Higashitsuji, Hiroshi Kashida, Tomohiro Watanabe, Yoriaki Komeda, Tomoyuki Nagai, Satoru Hagiwara, Masayuki Kitano, Naoshi Nishida, Takaya Abe, Hiroshi Kiyonari, Katsuhiko Itoh, Jun Fujita, Masatoshi Kudo

**Affiliations:** ^1^ Department of Gastroenterology and Hepatology, Kindai University Faculty of Medicine, Osaka-Sayama, Osaka, Japan; ^2^ Department of Clinical Molecular Biology, Kyoto University, Kyoto, Japan; ^3^ Genetic Engineering Team, RIKEN Center for Life Science Technologies, Kobe, Japan; ^4^ Animal Resource Development Unit, RIKEN Center for Life Science Technologies, Kobe, Japan

**Keywords:** IBD, TNF, Bmi1, IL-17, treatment resistance

## Abstract

Although long-standing colonic inflammation due to refractory inflammatory bowel disease (IBD) promotes the development of colitis-associated cancer (CAC), the molecular mechanisms accounting for the development of CAC remains largely unknown. In this study, we investigated the role of gankyrin in the development of CAC since gankyrin is overexpressed in sporadic colorectal cancers. We analyzed gene expression of colon tissues obtained from 344 patients with IBD and CAC and found that expression of gankyrin was much higher in colonic mucosa of patients with refractory IBD than in those with IBD in remission. Expression of gankyrin was upregulated in inflammatory cells as well as tumor cells in colonic mucosa of patients with CAC. Over-expressing studies utilizing tagged ganlyrin-cDNA identified physical interaction between ganlyrin and Src homology 2-containing protein tyrosine phosphatase-1 (SHP-1). Importantly, the interaction between ganlyrin and SHP-1 leads to inhibition of STAT3 activation and to enhancement of TNF-α and IL-17 in inflammatory cells. To further address the role of gankyrin in the development of CAC, we created mice with intestinal epithelial cell-specific gankyrin ablation (*Vil-Cre;Gankyrin^f/f^*) and deletion of gankyrin in myeloid and epithelial cells (*Mx1-Cre;Gankyrin^f/f^*). Gankyrin deficiency in myeloid cells, but not in epithelial cells, reduced the activity of mitogen activated protein kinase and the expression of stem cell markers, leading to attenuated tumorigenic potential. These findings provide important insights into the pathogenesis of CAC and suggest that gankyrin is a promising target for developing therapeutic and preventive strategies against CAC.

## INTRODUCTION

Colorectal cancer is one of the most leading causes of cancer-related death worldwide [1, 2]. Inflammatory bowel diseases (IBD); ulcerative colitis (UC) and Crohn's disease (CD) are thought to result from aberrant activation of the intestinal immune system [3] and are major risk factors for colorectal cancer, so-called colitis-associated cancer (CAC) [4]. Indeed, patients with IBD with refractory and longstanding colitis bear higher risk of colorectal cancer than individuals in the general population [5]. However, the exact molecular mechanisms underlying CAC remain unclear. In our previous reports, we found that stress response proteins induced in longstanding inflamed mucosa promote the development of CAC [6, 7]. In addition, we found that heat shock protein A4 (HSPA4) expression could predict poor therapeutic response to steroid in IBD patients. These data suggest that persistent inflammation can result in treatment resistance and refractory clinical course, then thereby increase the risk of CAC in IBD due to enhanced stress protein responses.

Gankyrin (also known as PSMD10, p28 and Nas6p) is an oncoprotein overexpressed in many malignancies including hepatocellular carcinoma, cholangiocellular carcinoma, colorectal cancer and lung cancer [8–12]. Given the fact that gankyrin enhances proliferation and death evasion in cancer cell lines [13–15], uncontrolled cell-autonomous events underlie the pathogenesis of cancer development through over-expression of ganlyrin. It should be noted, however, that the tumor microenvironment makes a major contribution and influences the physiology of malignant cells [16] in addition to cell-autonomous events such as proliferation and death evasion. At present, little has been understood regarding the involvement of gankyrin in inflammation-associated cancer.

Here we examined whether gankyrin plays a role in inflammation-associated tumorigenesis in the colon using a murine CAC model in which two kinds of tissue-specific gankyrin-deficient mice, intestinal epithelial cell-specific gankyrin ablation (*Vil-Cre;Gankyrin^f/f^*) mice and deletion of gankyrin in both myeloid and epithelial cells (*Mx1-Cre;Gankyrin^f/f^*) mice, were employed. We found that expression of gankyrin in myeloid cells rather than in epithelial cells is required for efficient tumorigenesis. Mechanistically, binding of gankyrin to SH2-containing protein tyrosine phosphatase-1 (SHP-1, also known as PTPN6) enhances the pro-inflammatory cytokine responses in myeloid cells through activation of signal transducer and activator of transcription-3 (STAT3) [17, 18] and then promotes colorectal tumorigenesis. Consistent with these results, refractory inflammation is associated with increased gankyrin expression in the colonic mucosa of patients with refractory IBD, which would increase the risk for CAC. Taken together, this study provides the evidence that gankyrin expression underlie the pathogenesis of inflammation-associated tumorigenesis.

## RESULTS

### Gankyrin expression is increased in the colonic mucosa of patients with refractory IBD

**Figure 1:**
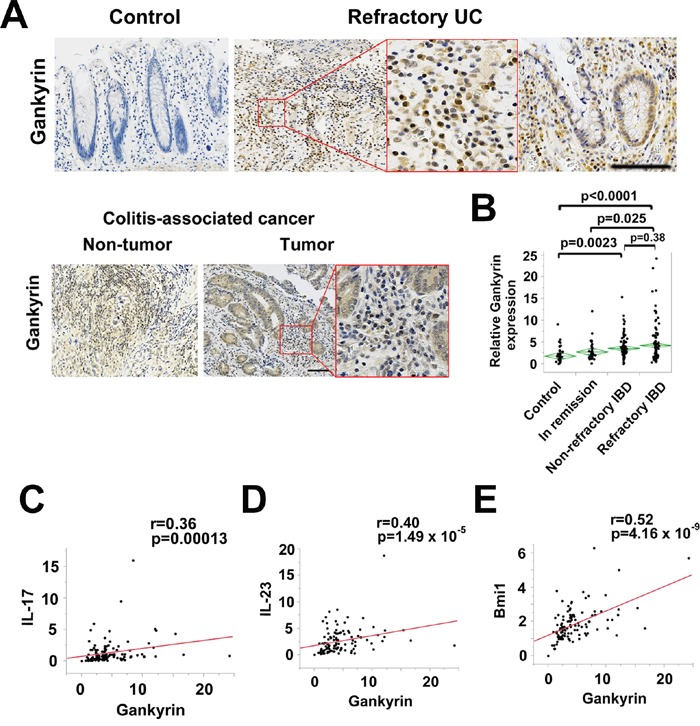
Gankyrin expression is increased in the colonic mucosa of patients with refractory IBD **A**. Representative images of immunohistochemical findings in human colonic mucosa of patients without IBD and those with refractory UC and human colitis-associated cancer (CAC) using anti-gankyrin antibody. Scale bar, 100 μm. **B**. Expression of gankyrin mRNA in normal colonic mucosa (Control, n = 54), colonic mucosa of IBD patients in remission (In remission, n = 47), those with non-refractory active IBD (Non-refractory, n = 115), and those with refractory IBD (Refractory, n = 128), as determined by quantitative real-time qPCR. *P* values were calculated by post-hoc Tukey-Kramer HSD multiple comparison. **C-E**. Scatter plot of relative mRNA levels of gankyrin and the respective genes in human colonic mucosa.

In our previous study, we showed that longstanding intestinal inflammation increases the expression of stress response proteins including HSPA4 in the colonic mucosa of IBD patients. Interestingly, such increased expression of HSPA4 was accompanied by that of gankyrin, which suggests possible involvement of ganlyrin in the development of long-standing colonic inflammation [7]. Based on these data, in this study we addressed whether an association exists between gankyrin expression and the clinical status of IBD patients. For this purpose, we obtained colonic biopsy specimens from patients with refractory IBD, non-refractory IBD, and IBD in remission. The disease characteristics were shown in Supplementary Table 1. Gankyrin expression levels were increased in patients with refractory IBD as compared with those in controls or IBD patients in remission (Figure 1A, 1B). By contrast, no significant difference was seen in gankyrin expression levels between non-refractory IBD patients and those in remission (Figure 1B), Serum albumin levels were significantly lower and Mayo endoscopic scores were significantly higher in non-refractory and refractory IBD patients than those in IBD patients in remission (Table S1). Thus, gankyrin expression was associated with the disease activity of IBD.

We then performed immunohistochemical studies to identify the cells expressing gankyrin in the human colon tissues. Gankyrin expression was enhanced in inflammatory cells as well as epithelial cells in chronically inflamed mucosa (Figure 1A). In addition, gankyrin expression was up-regulated in all human CAC cases (total n=10) and was detected in inflammatory cells as well as epithelial cells and tumor cells (Figure 1A). Taken together, these data suggest that expression of gankyrin is up-regulated in inflammatory cells, epithelial cells, and tumor cells in the colonic mucosa of patients with refractory IBD and CAC.

### Gankyrin expression is correlated with expression of pro-inflammatory cytokines and stem cell markers

It is generally accepted that colonic mucosa of patients with IBD is characterized by pro-inflammatory cytokine responses. Th17 pathways induced by myeloid cell production of IL-6 and IL-23 have been implicated in the immuno-pathogenesis of IBD [19]. Therefore, we next assessed the relationship between pro-inflammatory Th17 responses and gankyrin expression in colonic mucosa of IBD. To this end, expression levels of IL-6, IL-23p19, and IL-17A were determined. Gankyrin expression correlated weakly but significantly with IL-6 expression with a linear coefficient of 0.26 in the colonic mucosa of UC patients but not of CD patients (Supplementary Figure 1A-1B). A significant correlation was seen between *Gankyrin* and *IL-17A* or *IL-23p19* mRNA expression in UC patients (Figure 1C–1D). Thus, it is likely that gankyrin could act in concert with pro-inflammatory Th17 responses to cause chronic colonic inflammation.

Having obtained positive correlation between pro-inflammatory cytokine responses and ganlyrin, we next turned our attention to a linkage between expression levels of gankyrin and stem cell markers. Leucine-rich repeat-containing G-protein-coupled receptor 5 (Lgr5) is a prototypical marker of the self-renewing multipotent adult stem cell populations residing in intestinal crypts that mediate regeneration of the intestinal epithelium [20]. Lgr5 expressed in intestinal stem cells has also been shown to self-renew and continuously replenish tumor progeny [21]. B cell-specific Moloney murine leukemia virus insertion site 1 (Bmi1) is frequently overexpressed in human sporadic colorectal cancer and the degree of upregulation correlates with disease progression and is predictive of poor patient survival [22]. In mice, Bmi1 is required for intestinal tumorigenesis [23]. In UC patients, a significant correlation was found between Lgr5 and Bmi1 expression (Supplementary Figure 1C). Interestingly, gankyrin expression significantly correlated with Bmi1 and Lgr5 expression with linear coefficients of 0.52 and 0.27, respectively in UC and correlated with Bmi1 expression in CD (Figure 1E and Supplementary Figure 1D–1E). Although gankyrin expression was shown to correlate with stemness factor Nanog expression in human sporadic colorectal cancer in previous study [10], no significant correlation was found between the expression of these genes in IBD patients (Supplementary Figure 1F). This positive correlation between gankyrin and Lgr5 or Bmi1 suggests that gankyrin might be related with cancer stem cell behavior in IBD patients.

### Gankyrin deficiency attenuates tumorigenesis in the murine CAC model

**Figure 2:**
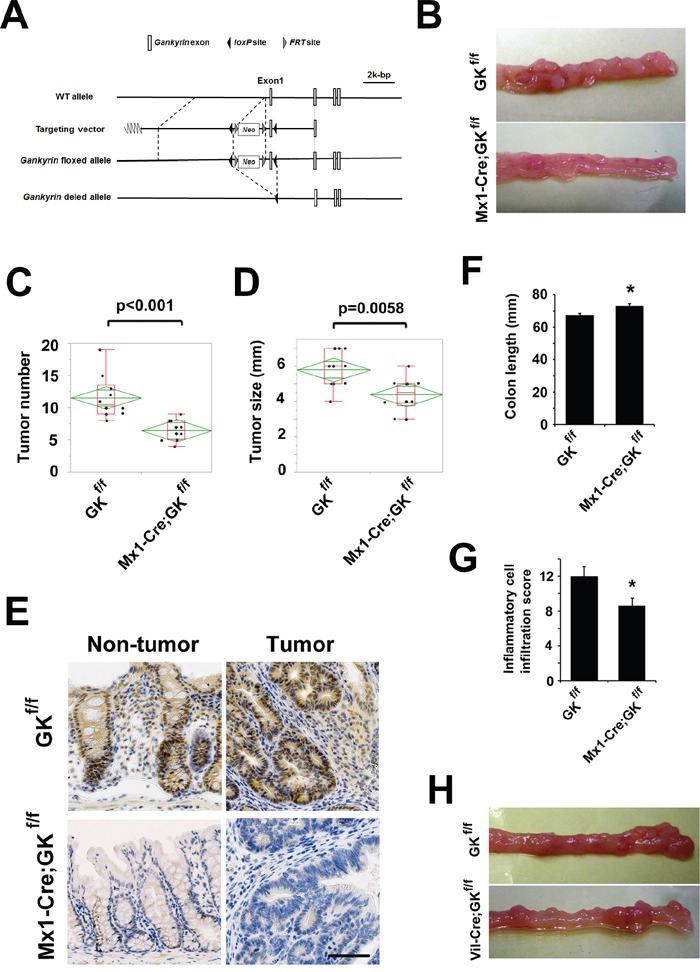
Gankyrin deficiency in myeloid cells attenuates tumorigenesis in the murine CAC model **A**. Construction of the wild-type allele, targeting vector, floxed allele and deleted allele of the mouse *Gankyrin* gene. The targeting vector contained the neo^r^ cassette for selection, and *loxP* fragments were located on both sides of exon 1 to delete this exon. **B**. Typical examples of macroscopic tumorigenesis in the CAC model. *Gankyrin^f/f^* (GK^f/f^) mice and *Mx1-Cre;Gankyrin^f/f^* (Mx1-Cre;GK^f/f^) mice were challenged with AOM and DSS, and colons were cut longitudinally. **C, D**. Tumor number (C) and maximum size (D, GK^f/f^ mice, n = 10; Mx1-Cre;GK^f/f^ mice, n = 10). **E**. Mx1-Cre;GK^f/f^ mice and GK^f/f^ mice were challenged with AOM and DSS. Representative images of immunohistochemical detection of gankyrin are shown. Scale bar, 100 μm. **F**. Colon length after treatment with AOM and DSS. GK^f/f^ mice, n = 6; Mx1-Cre;GK^f/f^ mice, n = 6. Data are means ± SEM. *P < 0.05 compared with GK^f/f^ mice. **G**. Inflammatory cell infiltration into colonic tissues of GK^f/f^ mice and Mx1-Cre;GK^f/f^ mice 7 days after the initiation of DSS administration. GK^f/f^ mice, n = 5; Mx1-Cre;GK^f/f^ mice, n = 5. Data are means ± SEM. *P < 0.05 compared with GK^f/f^ mice. **H**. Typical examples of macroscopic tumorigenesis in the CAC model. GK^f/f^ mice and *Villin-Cre;Gankyrin^f/f^* (Vil-Cre;GK^f/f^) mice were challenged with AOM and DSS, and colons were cut longitudinally.

Chronic inflammation increases intestinal cancer risk in IBD [24]. To investigate the precise pathogenic mechanisms underlying IBD-associated colorectal carcinogenesis, we used the AOM plus DSS mouse model to study the role of gankyrin in CAC. Since gankyrin expression was up-regulated in a wide variety of cells including immune cells, epithelial cells, and tumor cells in human IBD and CAC samples, we initially tried to determine the role of gankyrin in the AOM-DSS mouse model. To this end, we created *Gankyrin^f/f^* mice (Figure 2A) and then crossed with Mx1-Cre mice to generate Mx1-Cre-driven gankyrin deficient mice (termed *Mx1-Cre;Gankyrin^f/f^* mice).

In the AOM/DSS protocol, a significant decrease was noted in the number and maximum size of tumors in the *Mx1-Cre;Gankyrin^f/f^* mice compared with *Gankyrin^f/f^* controls (Figure 2B–2D). These tumors were located in the middle to distal colon, which is similar to human CAC. In *Gankyrin^f/f^* mice, gankyrin expression was detected in both epithelial and in lamina propria immune cells of non-tumor colonic tissue by immuno-histochemical analysis. In the colonic tumor lesions, both tumor cells and surrounding stromal cells expressed gankyrin protein (Figure 2E). Gankyrin expression was much lower both in myeloid and epithelial cells of non-tumor colonic tissue of *Mx1-Cre;Gankyrin^f/f^* mice as compared with *Gankyrin^f/f^* controls. Gankyrin was absent in most of tumors of *Mx1-Cre;Gankyrin^f/f^* mice; however, a few tumors expressed gankyrin protein (Figure 2E and Supplementary Figure 2A). Thus, these data utilizing the AOM-DSS model suggest that gankyrin expression in myeloid cells and/or epithelial cells is associated with the development of CAC.

We next assessed the effects of gankyrin deletion on the chronic inflammatory responses in this model. Colon length was measured as one parameter to evaluate the severity of inflammation and was found to be significantly longer in *Mx1-Cre;Gankyrin^f/f^* mice than in *Gankyrin^f/f^* mice (Figure 2F). Pathological analysis of non-tumor portions showed a marked infiltration of immune cells in *Mx1-Cre;Gankyrin^f/f^* mice as compared with *Gankyrin^f/f^* mice (Supplementary Figure 2B). No differences in body weight could be detected between *Mx1-Cre;Gankyrin^f/f^* mice and *Gankyrin^f/f^* mice during the CAC regimen (Supplementary Figure 2C).

To assess the effect of gankyrin on acute inflammation, experimental colitis was induced by treating mice with 2.5% DSS for 7 days. The degree of inflammatory cell infiltration into the colon was lower in *Mx1-Cre;Gankyrin^f/f^* mice than in *Gankyrin^f/f^* mice (Figure 2G and Supplementary Figure 3A). Apoptosis detected by TUNEL staining was observed primarily in the colonic crypts and was not affected by gankyrin disruption (Supplementary Figure 3B). In addition, no significant difference in body weight was found during the seven-day colitis regimen (Supplementary Figure 3C). These data altogether suggest that loss of gankyrin expression in hematopoietic and non-hematopoietic cells leads to the prevention of CAC development through the attenuation of inflammatory responses.

### Gankyrin in myeloid cells is critical for efficient tumorigenesis in the murine CAC model

To functionally characterize the contribution of different cell populations to colitis development, we created bone marrow-chimeric mice using a combination of gamma irradiation and bone marrow transplantation (BMT). Non-transplanted irradiated mice survived less than 2 weeks after irradiation, indicating successful deletion of BM cells by gamma irradiation. Irradiated animals that received BMT were allowed to recover for 2 months prior to placing them on the DSS-induced colitis protocol. Inflammatory cell infiltration scores of irradiated gankyrin-intact mice harbouring gankyrin-deficient BM cells was lower than those harbouring gankyrin-intact BM cells (Supplementary Figure 3D), which indicates that gankyrin expressed in hematopoietic cells is involved in the attenuated inflammation observed in *Mx1-Cre;gankyrin^f/f^* mice. To address the therapeutic ability of gankyrin inhibition, we injected poly(I:C) into *Mx1-Cre;Gankyrin^f/f^* mice and deleted gankyrin after the initiation of DSS treatment. Inflammation was slightly attenuated by gankyrin deletion after the DSS treatment (Supplementary Figure 3E). Consistent with the data shown above, flow-cytometric analysis revealed that CD3^+^ T cells, CD11b^+^ myeloid cells, and CD20^+^ B cells were positive for intracellular gankyrin expression (Supplementary Figure 4A).

In *Mx1-Cre;Gankyrin^f/f^* mice, gankyrin was deleted in the liver as well. To confirm the role of gankyrin expression in the colon, we created *Albumin-Cre;gankyrin^f/f^* mice where gankyrin is deficient only in the liver (Sakurai et al., unpublished data) and compared *Albumin-Cre;gankyrin^f/f^* mice and *gankyrin^f/f^* mice in colitis model. No difference in DSS-induced colonic inflammation was seen between *Albumin-Cre;gankyrin^f/f^* mice and *gankyrin^f/f^* mice (Supplementary Figure 4B), suggesting that gankyrin deficiency in the colon, but not the liver, affects the phenotype observed in DSS-challenged *Mx1-Cre;gankyrin^f/f^* mice.

Mice lacking gankyrin only in their intestinal epithelial cells (*Vil-Cre;Gankyrin^f/f^* mice) and their littermate controls (*Gankyrin^f/f^* mice) were challenged with AOM and DSS to confirm a role of myeloid cells expressing gankyrin. Deletion of gankyrin in enterocytes did not show gross abnormalities. No significant differences were seen in tumor numbers or maximum sizes between *Vil-Cre;Gankyrin^f/f^* mice and control *Gankyrin^f/f^* mice in the murine CAC model (Figure 2H and Supplementary Figure 5A-5B). Collectively, these data suggest that gankyrin promotes tumorigenesis mainly through its actions in myeloid cells rather than epithelial cells.

### Gankyrin interacts with SHP-1 in myeloid cells

**Figure 3:**
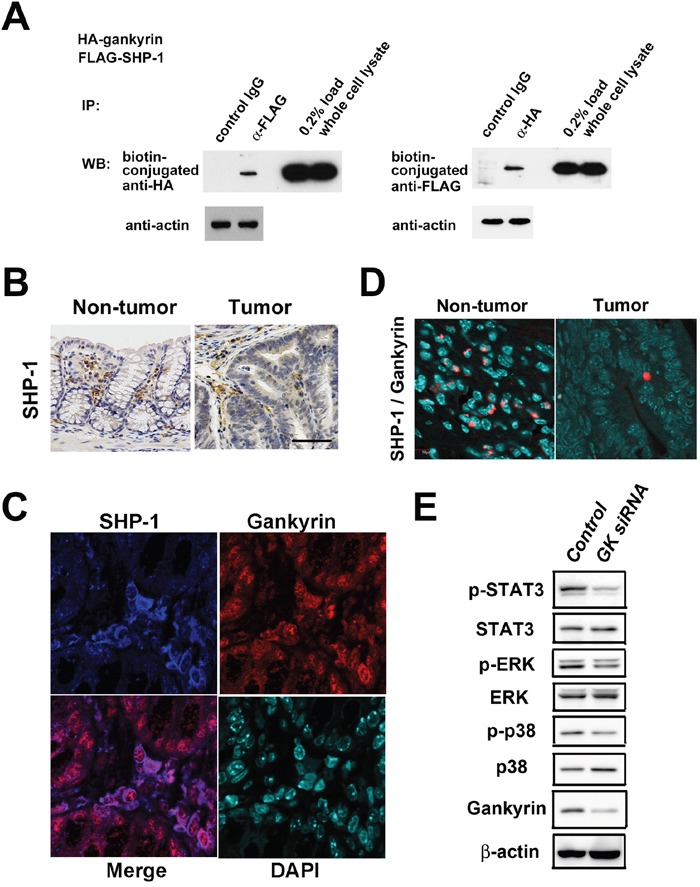
Gankyrin interacts with SHP1 *in vitro* and *in vivo* **A**. Coimmunoprecipitation (IP) of exogenous proteins. U-2 OS cells were cotransfected with plasmids expressing HA-gankyrin and FLAG-SHP-1. Immunoprecipitates prepared by IP with indicated antibodies were analyzed by western blotting (WB). **B**. Representative images of immunohistochemical detection of SHP-1 in colonic tissues and tumors of *Gankyrin^f/f^* mice treated with AOM and DSS. Scale bar, 100 μm. **C**. Representative images of immunohistochemical detection of SHP-1 and gankyrin in colonic tissues from tumor-harboring *Gankyrin^f/f^* mice. **D**. Interaction of gankyrin with SHP-1 was assessed by Duolink Assay in AOM/DSS-treated *Gankyrin^f/f^* mice. **E**. Lysates from THP-1 cells transfected with *gankyrin* RNAi (GK siRNA) or control RNAi (Control) were analyzed by WB using indicated antibodies.

To further characterize the tumorigenic activity of gankyrin, we performed a yeast two-hybrid assay using the full length of gankyrin as bait, and found that gankyrin bound to SHP-1 in the yeast. To confirm that gankyrin interacts with SHP-1 in mammalian cells, U-2 OS cells were co-transfected with plasmids expressing HA-tagged human gankyrin and FLAG-tagged SHP-1. When cell lysates were immunoprecipitated with an anti-FLAG antibody, HA-gankyrin was detected in the precipitates. Reciprocally, SHP-1 was detected in the anti-HA immunoprecipitates from cells co-transfected with plasmid expressing HA-gankyrin and FLAG-SHP-1 (Figure 3A). Thus, a physical interaction between gankyrin and SHP-1 was confirmed in over-expression studies.

We next addressed a physioligical role played by the interaction between ganlyrin and SHP-1 in AOM-DSS model. In *Gankyrin^f/f^* mice challenged with AOM and DSS, SHP-1 was expressed mainly in inflammatory or stromal cells as well as tumor cells (Figure 3B). Double immunofluorescence staining showed that both gankyrin and SHP-1 proteins were co-localized in the cytoplasm and the nucleus of these cells (Figure 3C). Finally, we employed, the Duolink Assay for the visulalization of interaction between gankyrin and SHP-1. The molecular interaction between ganlyrin and SHP-1 visualized as red punctate dots were mainly seen in non-epithelial cells, but not epitherlila cells (Figure 3D). These data suggest that the molecular interaction between gankyrin and SHP-1 is operating in non-epithelila cells i.e., hematopoietic myleloid cells, in AOM-DSS model.

### Interaction of gankyrin with SHP-1 results in activation of STAT3 and MAP kinase

SHP-1 is well-known inhibitor of activation-promoting signaling cascades in hematopoietic cells [25] and negatively regulates signaling by dephosphorylating STAT3, ERK, JNK and p38 [17, 18, 26, 27]. Having obtained the induction of gankyrin-SHP1 interaction in hematopoietic cells, we next assessed the effects of such interaction on these signaling pathways in myeloid cells. To this end, we utilized THP-1 cells, an immortalized line of human monocyte. Knockdown of gankyrin expression by ganlyrin-specific siRNA reduced STAT3 and p38 phosphorylation in immuno-blotting analysis (Figure 3E). Thus, gankyrin promotes activation of STAT3 and mitogen- activated protein kinase (MAP kinase). In colon tumor cells, knockdown of gankyrin reduced cell viability while knockdown of SHP-1 increased it (Supplementary Figure 6A). In contrast, overexpression of gankyrin did not affect SHP-1 phosphatase activity probably because of much endogenous gankyrin protein (Supplementary Figure 6B).

**Figure 4:**
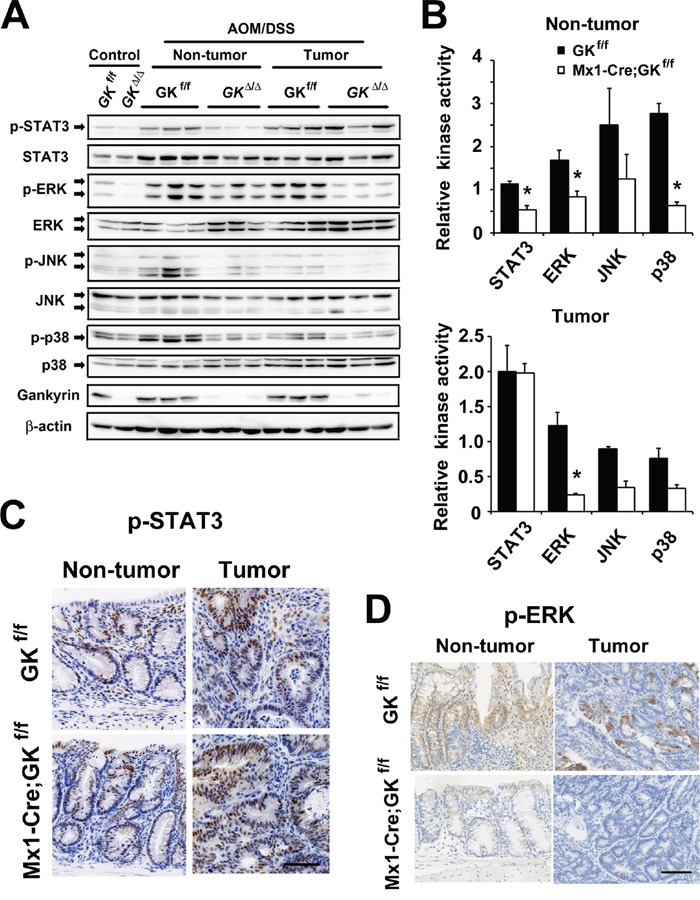
Gankyrin activates STAT3 in non-tumor tissues and ERK in tumors **A**. *Mx1-Cre;Gankyrin^f/f^* (Mx1-Cre;GK^f/f^) mice and *Gankyrin^f/f^* (GK^f/f^) mice were challenged with AOM and DSS. Homogenates of non-treated colons (Control), non-tumor colon tissues (Non-tumor) and tumors (Tumor) were gel-separated and immunoblotted with the indicated antibodies. **B**. Relative kinase activities in non-tumor colon tissues (Non-tumor) and tumors (Tumor) isolated from *Mx1-Cre;Gankyrin^f/f^* (Mx1-Cre;GK^f/f^) mice and *Gankyrin^f/f^* (GK^f/f^) mice challenged with AOM and DSS were assessed by densitometry. Kinase activity in untreated *Gankyrin^f/f^* mice was given an arbitrary value of 1. Data are means ± SEM. **C, D**. Representative images of immunohistochemical detection of phosphorylated STAT3 (C) and phosphorylated ERK (D) in non-tumor colon tissues (Non-tumor) and tumors (Tumor). Scale bar, 100 μm.

Then, we assessed the effects of gankyrin on the signaling pathways *in vivo*. Consistent with the data obtained from human THP-1 cells, *Mx1-Cre;Gankyrin^f/f^* mice exhibited reduced activation of STAT3, ERK and p38 compared with *Gankyrin^f/f^* controls (Figure 4A and 4B). Immunohistochemical analysis revealed that the activities of STAT3 and ERK were reduced in inflammatory cells but not in intestinal epithelial cells in gankyrin-deficient colons (Figure 4C and 4D). In contrast, tumors isolated from *Mx1-Cre;Gankyrin^f/f^* mice showed similar level of phosphorylated STAT3 and lower level of phosphorylated ERK compared with those from control *Gankyrin^f/f^* mice (Figure 4A, 4B and 4D). Colorectal tissues isolated from *Vil-Cre;Gankyrin^f/f^* mice exhibited similar level of phosphorylated STAT3 and ERK compared with control counterparts (Supplementary Figure 5C). Thus, these data suggest that gankyrin deletion leads to reduced activation of STAT3 and MAP kinase in inflammatory myeloid cells rather than epithelial cells in AOM-DSS model.

We assessed the effect of gankyrin on these signaling pathways in DSS-induced colitis model. Examination of the colonic lysates from DSS-treated *Villin-Cre;Gankyrin^f/f^* mice and *Gankyrin^f/f^* controls showed that gankyrin deletion resulted in reduced STAT3 phosphorylation but a minor change in ERK phosphorylation (Supplementary Figure 7A), whcih suggests a possibel involvement of gankyrin-SHP1 interaction in the activation of STAT3 and MAP kinase in acute epithelial cell injury.

### Loss of gankyrin in myeloid cells results in reduced expression of cytokines and stem cell markers

**Figure 5:**
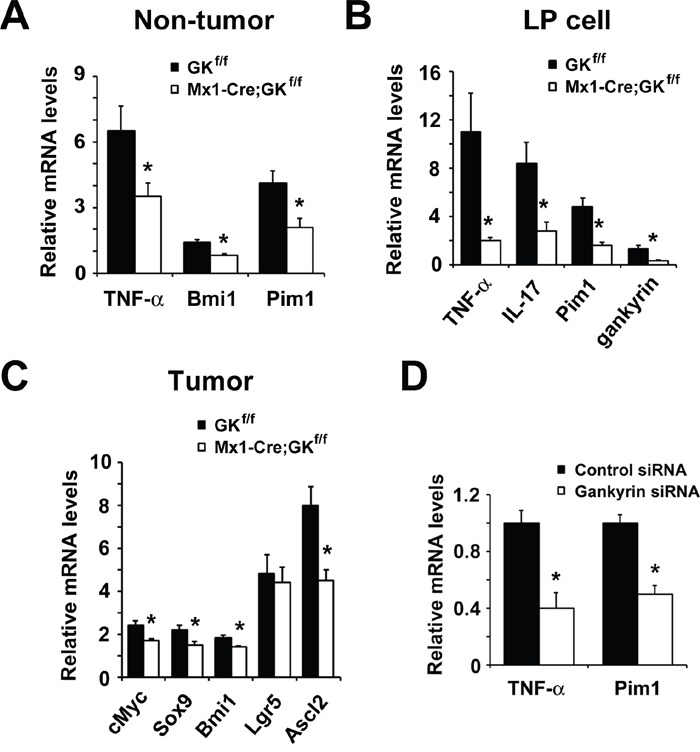
Loss of gankyrin in myeloid cells results in reduced expression of cytokines and stem cell markers **A**. RNA was extracted from non-tumor colon of tumor-harboring *Mx1-Cre;Gankyrin^f/f^* (Mx1-Cre;GK^f/f^) mice and *Gankyrin^f/f^* (GK^f/f^) mice. Relative amounts of mRNA were determined by real-time qPCR and normalized to the amount of actin mRNA. The amount of each mRNA in the untreated colon was given an arbitrary value of 1.0. Data are means ± SEM (n = 5). **B**. Effect of gankyrin on gene expression in colonic lamina propria (LP) cells. LP cells were isolated from DSS-treated *Mx1-Cre;Gankyrin^f/f^* (Mx1-Cre;GK^f/f^) mice and *Gankyrin^f/f^* (GK^f/f^) mice and cytokine mRNA expression was analyzed by real-time qPCR. The mRNA expression levels in LP cells from non-treated WT mice were set as 1. **C**. RNA was extracted from tumor tissues of *Mx1-Cre;Gankyrin^f/f^* (Mx1-Cre;GK^f/f^) mice and *Gankyrin^f/f^* (GK^f/f^) mice. Relative amounts of mRNA of ERK target genes cMyc, and stem cell markers Sox9, Bmi1, Lgr5 and Ascl2 were determined by real-time qPCR and normalized to the amount of actin mRNA. The amount of each mRNA in the untreated colon was given an arbitrary value of 1.0. Data are means ± SEM (n = 5). **D**. Relative amounts of mRNA of TNF-α and Pim1 from THP-1 cells transfected with *gankyrin* RNAi or control RNAi were determined by real-time qPCR and normalized to the amount of actin mRNA. The amount of each mRNA in the untreated colon was given an arbitrary value of 1.0. Data are means ± SEM (n = 3).

The associated immune response was investigated by analyzing colonic cytokine levels. Colonic tissue from *Mx1-Cre;Gankyrin^f/f^* mice showed lower levels of pro-inflammatory cytokine TNF-α than that of control mice (Figure 5A) as assessed by qPCR analysis, which would be due to reduced activities of STAT3 and MAP kinase in inflammatory cells. To further characterize the effects of gankyrin on cytokine production, we sought to confirm these findings *in vitro*. In LP cells isolated from DSS-treated colons of *Mx1-Cre;Gankyrin^f/f^* mice, expression of TNF-α and IL-17 was decreased compared with those of control mice (Figure 5B), which is consistent with the data in humans (Figure 1C).

It is genellay accepted that pro-inflammatory cytokine responses affect the expression of stem cell markers and proliferation of stem cells [28]. Therefore, we addressed the effects of gankyrin deletion on the expression of cancer stem cell markers. Colonic tissue from *Mx1-Cre;Gankyrin^f/f^* mice showed lower levels of Bmi1 than that of *Gankyrin^f/f^* mice when they were treated with AOM and DSS (Figure 5A). In contrast, no difference was found in the level of Bmi1 between similarly treated *Vil-Cre;Gankyrin^f/f^* mice and *Gankyrin^f/f^* mice (Supplementary Figure 5D).

In *Mx1-Cre;Gankyrin^f/f^* mice, STAT3 activity was decreased in inflammatory cells whilst ERK activity was decreased in tumor cells as well as inflammatory cells. Provirus integration site for Moloney murine leukemia virus (Pim1) is linked to the development and progression of several cancers including colorectal cancer [29]. Sox9 [sex-determining region Y (SRY)-box 9 protein], an intestinal stem cell marker, plays critical roles during embryogenesis and its activity is required for development, differentiation, and lineage commitment in various tissues including the intestinal epithelium [30]. Since expression of Pim1 and Sox9 requires activation of STAT3 and ERK, respectively, we assessed the expression of Pim1 and Sox9 in AOM-DSS model utilizing conditional gankyrin–deficient mice. Expression of Pim1 was down-regulated in gankyrin-deficient colons compared to control counterparts (Figure 5A). Furthermore, LP cells derived from *Mx1-Cre;Gankyrin^f/f^* mice, but not from *Vil-Cre;Gankyrin^f/f^* mice, exhibited marked down-regulation of *Pim1* mRNA relative to those isolated from *Gankyrin^f/f^* mice (Figure 5B and Supplementary Figure 7B). *Sox9* mRNA expression was significantly reduced in gankyrin-deficient tumors (Figure 5C). Another stem cell marker achaete-scute complex homolog 2 (Ascl2) is suggested to regulate the development of colorectal cancer via CDX2 [31]. Gankyrin ablation also decreased the expression of Ascl2 (Figure 5C). In addition, expression of cMyc, a prototypical oncogene, was reduced in gankyrin-deficient mice as compared with gankyrin-intact mice (Figure 5C). Taken together, these data support the idea that gankyrin deletion leads to a reduction of pro-inflammatory cytokine responses and expression of cancer stem cell markers through inhibition of STAT3 and/or ERK activation.

In THP-1 cells, an immortalized line of human monocyte, knockdown of gankyrin reduced the expression of TNF-α and Pim1 (Figure 5D) through the inhibition of p38 and STAT3 activation (Figure 3E). In contrast, the effect of gankyrin on ERK activation was marginal in THP-1 cells (Figure 3E). These data indicate that gankyrin activates STAT3 in a cell-autonomous manner, leading to increased expression of their target genes such as TNF-α, which might at least partially contribute to the enhanced MAP kinase ERK activation in a non-cell-autonomous manner.

## DISCUSSION

In the context of chronic inflammation, cytokines secreted by immune cells contribute to creating the cancer-microenvironment in which activation and proliferation of cancer stem cells are promoted [28]. Indeed, a recent study has suggested that colorectal cancer tissue-derived Foxp3^+^ IL-17^+^ cells have the capacity to induce cancer-initiating cells *in vitro* [32]. Thus, it is generally accepted that cytokine-mediated inflammatory signaling pathways are important for dedifferentiation and generation of tumor-initiating cells [33]. However, the precise mechanisms accounting for the activation of cancer-initiating cells in response to pro-inflammatory cytokines have been poorly defined. In this study, we provide the evidence that gankyrin expressed in myeloid cells promote the expansion of cancer-initiating cells through the induction of cancer stem cell markers such as Pim1 and Sox9. As for the mechanisms of ganlyrin-induced inflammatory responses by myeloid cells, we show that molecular interaction between gankyrin and SHP1 leads to secretion of pro-inflammatory cytokine. Thus, we have elucidated a part of molecular mechanisms for the development of inflammation-associated colon carcinogenesis by focusing on the function of myeloid cell expression of gankyrin.

Previous studies report that upregulation of gankyrin was detected in many tumor cell lines and primary tumors. Such enhanced expression of gankyrin has been considered to promote the prolifearion of cancer cells by inhibiting apoptosis and cell cycle in cell lines [8–14]. However, until now it was not clear whether gankyrin is instrumental for tumor development *in vivo*. In this study, we tried to determine the role of gankyrin in the interface between tumor cells and immune cells. We found that gankryin upregulates IL-17 expression in lamina propria cells in murine AOM-DSS CAC model. In addition, we showed that expression of gankyrin was positively correlated with that of IL-17A. Recent study by Wang K et al. show that IL-17A exerts its pro-tumorigenic activity through its type A receptor (IL-17RA), which signals directly within transformed colonic epithelial cells to promote early tumor development by activating MAP kinase, especially ERK signaling [34]. Consistent with this report, a significant reduction of IL-17 production was seen in the colonic mucosa of gankyrin-deficient mice upon AOM-DSS treatment, which effect was accompanied by a reduction in MAP kinase activation and in expression of cancer stem cell markers. Thus, IL-17 signaling might mediate the gankyrin-mediated cross-talk between immune cells and tumor cells.

Another pro-inflammatory cytokine associated with the development of CAC through activation of gankyrin is TNF-α. Expression of TNF-α was positively correlated with that of gankyrin in human IBD samples. In addition, TNF-α expression was significantly lower in gankyrin-deficient mice treated with AOM-DSS than in gankyrin-intact mice. Furthermore, ganlyrin-knowdown leads to a diminished TNF-α production by human monocytic cells through inhibition of STAT3 and MAP kinase activation, which suggests involvement of gankyrn-mediated signaling pathways for optimal production of TNF-α. Given the fact that TNF-α produced by myeloid cells is a critical player for the longstanding chronic inflammation and CAC [35], the results of the present study provide another important linkage between gankyrin-mediated TNF-α production and colon carcinogenesis.

In this study, we created two kinds of conditional gankyrin-deficient mice; *Mx1-Cre;Gankyrin^f/f^* mice and *Vil-Cre;Gankyrin^f/f^* mice. Deletion of gankyrin was confirmed in hemapoietic and non-hematopoietic cells in *Mx1-Cre;Gankyrin^f/f^* mice whereas deletion was confirmed in epithelial cells in *Vil-Cre;Gankyrin^f/f^* mice. The development of CAC induced by AOM-DSS treatment was significantly attenuated in the colon of *Mx1-Cre;Gankyrin^f/f^* mice as compared with control *Gankyrin^f/f^* mice. In contrast, the development of CAC induced by AOM-DSS treatment was comparable between *Vil-Cre;Gankyrin^f/f^* mice and control *Gankyrin^f/f^* mice. Similarly, *Mx1-Cre;Gankyrin^f/f^* mice exhibited attenuated inflammatory scores upon acute DSS colitis model. In line with this, the development of acute DSS colitis was attenuated in irradiated-gankyrin-intact mice transplanted gankyrin-deficient BM cells as compared with thoses transplanted with gankyrin-intact BM cells. These studies utilizing two kinds of conditional gankyrin-deficient mice together with BMT experiments strongly suggest a pivotal role played by hematopoietic cells expressing gankyrin in the development of chronic colitis and colitis-associated cancer. Compatible to this idea, gankyrin expression was detected in immune cells of the colon tissue of human CAC and refractory IBD samples. Moreover, pro-inflammatory cytokine responses such as TNF-α and IL-17 were significantly reduced in the colon tissues of AOM-DSS-treated *Mx1-Cre;Gankyrin^f/f^* mice, which effects were again accompanied by a reduction in expression of cancer stem cell markers and in activation of STAT3 and MAP kinase activation. Thus, it is very likely that myeloid cells expressing gankyrin play an indispensable role in the development of chronic colitis and CAC through the enhancement of pro-inflammatory cytokine responses, activation of STAT3 and MAP kinases, and finally expression of cancer stem cell markers. Therefore, present findings show for the first time that as a tumor promoter gankyrin can enhance tumor development through the interaction between tumor cells and immune cells. It should be noted, however, that we cannot exclude a role played by gankyrin-expressing epithelial cells. Gankyrin in epithelial cells would at least partly contribute to tumorigenesis in another type of cancer such as sporadic colorectal cancer rather than CAC.

One question arising from the present study is whether gankyrn expression is up-regulated not only in IBD but also in non-chronic inflammatory diseases such as infectious or ischemic colitis in human samples. In this regards, we do not have enough specimens of non-chronic inflammatory diseases and thus cannot provide the data of gankyrin expression in non-chronic inflammatory diseases. However, our preliminary assay utilizing biopsy samples from Amoebatic dyscentery revealed that the gankyrin expression was found to be lower than that in IBD patients.

The most feared long-term complication of IBD is CAC as patients with IBD have an increased risk of colorectal cancer [5]. Most cases of CAC arise from dysplasia, and surveillance colonoscopy is therefore recommended to detect dysplasia. Recently, it was suggested that intensified surveillance and endoscopic resection of dysplasia might prevent CAC [36], which is a new strategy to improve quality of life for patients. If we can reliably predict an individual's risk of dysplasia and CAC, surveillance strategies could be appropriately personalized and surveillance programs would become more cost-effective. As shown in the present study, high level of gankyrin expression in the colonic mucosae reflects the presence of refractory inflammation. This suggests that gankyrin can be used as a potential marker for predicting the risk of CAC development. Indeed, expression of gankyrin was increased in human CAC tissues as well. Furthermore, gankyrin expression was reported to be upregulated during the development of another type of cancer [15, 37]. Analyzing the gankyrin level may be utilized to predict the risk of cancer and prognosis of IBD patients.

In this study, we propose that gankyrin expressed in myeloid cells promotes CAC through induction of pro-inflammatory cytokine responses and expression of cancer stem cell markers. The chain of evidence supporting this conclusion consisted first of the fact that gankyrin expression is correlated with the expression of immune cell-derived pro-inflammatory IL-17-related cytokines and with that of stem cell marker (Lgr5) in the colonic mucosa of IBD patients. Moreover, immuno-histochemical analysis revealed the expression of gankyrin in immune cells migrated into the colonic mucosa of patients with refractory IBD and CAC. These results obtained from human samples were further corroborated in experimental studies utilizing *Mx1-Cre;Gankyrin^f/f^* mice and *Vil-Cre;Gankyrin^f/f^* mice. As mentioned above, *Mx1-Cre;Gankyrin^f/f^* mice lacking gankyrin in hematopoietic cells and epithelial cells, but not *Vil-Cre;Gankyrin^f/f^* mice lacking gankyrin in epithelial cells alone, were resistant to the development of acute colitis and inflammation-associated colon tumorigenesis. Importantly, such resistance to inflammation-associated colon tumorigenesis in *Mx1-Cre;Gankyrin^f/f^* mice was associated with a significant reduction of pro-inflammatory responses (IL-17 and TNF-α) and expression of cancer stem cell markers (Bmi1 and Sox9). A final step in the chain of evidence consisted of yeast-two hybrid, over-expression, and gene-silenecing studies showing that gankyrin interacts with SHP-1 to induce activation of STAT3 and MAP kinases and then to induce TNF-α production. In accordance with this, a physical interaction between gankyrin and SHP-1 was detected in immune cells of the colon tissue of gankyrin-intact mice treated with AOM and DSS. Collectively, these data strongly suggest that gankyrin-expressing myeloid cells produce pro-inflammatory cytokines through its interaction with SHP-1 followed by STAT3 activation and then promote CAC through induction of stem cell markers. Thus, we propose gankyrin as a tumor promoter that plays a role in the interface between immune cells and epithelial cells. It should be noted, however, that confirmation of this notion awaits future studies addressing the relationship between gankyrin expression and colon cancer prognosis as well as those addressing the role of gankyrin in immune cells by using myeloid, T cell, or B cell-specific gankyrin deletion mice.

**Figure 6:**
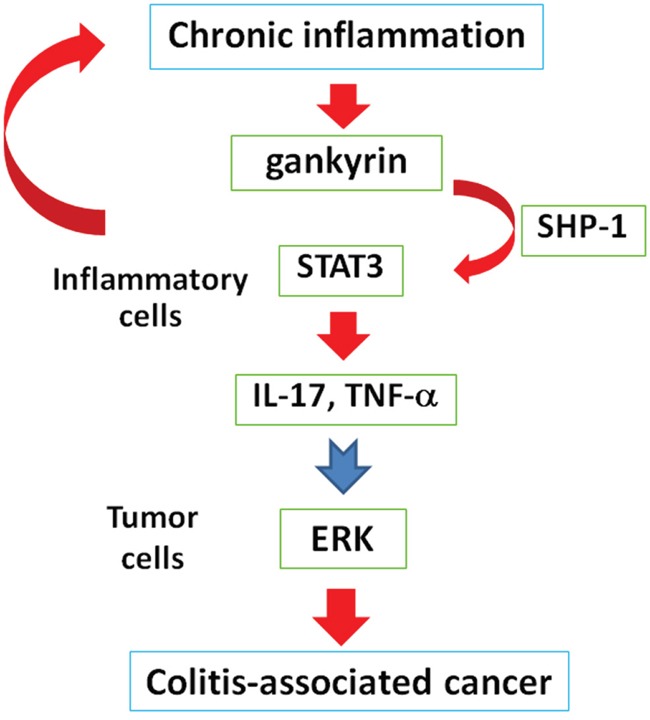
Chronic inflammation increases gankyrin expression in the colonic mucosa of patients with IBD In inflammatory cells, gankyrin activates STAT3 by binding to SHP-1, leading to enhanced inflammation. These augmented inflammatory responses would activate ERK in tumors and eventually promote colitis-associated cancer.

Taken together, gankyrin, whose expression is upregulated by chronic inflammation, increases STAT3 activity and the production of TNF-α and IL-17 by binding to SHP-1 in inflammatory cells, leading to augmented inflammation. Such pro-inflammatory cytokine responses may enhance MAP kinase activity in the tumor and upregulate expression of stem cell markers in the colon. These factors would eventually promote CAC (Figure 6). Thus, suppression and measurement of gankyrin expression is a promising approach for advanced treatment and personalized management of IBD patients.

## PATIENTS AND METHODS

### Human tissue samples

UC and CD were diagnosed as previously described [38]. In total, 344 intestinal mucosa specimens were obtained by endoscopy from UC patients including 109 cases of refractory UC, 106 cases of non-refractory active UC, 40 cases in remission, as well as 35 CD patients and 54 normal controls without IBD and gankyrin expression was assessed. Among 344 intestinal mucosa specimens, 111 intestinal samples were obtained between April 2011 and March 2013 and analyzed for the expression of several genes. CAC specimens were obtained from 10 patients who had undergone surgery. Refractory IBD was defined according to endoscopic criteria and categorized as being active for more than 6 months. Active inflammation was defined as Mayo endoscopic score ≥2 in UC cases and a presence of ulcer in CD cases or presence of symptoms. The Mayo score was previously described [39]. The clinical study protocol conformed to the ethical guidelines of the 1975 Declaration of Helsinki and was approved by the relevant institutional review boards.

### Generation of mice with tissue-specific deletion of the gankyrin gene

Targeted ES clones were isolated from HK3i ES cells [40]. Chimeric mice were generated as described [41] and crossed with C57BL/6 mice to obtain heterozygous *Gankyrin^flox/wt^* mice (Accession No. CDB0944K) [42]; *Gankyrin^flox/wt^*, *Mx1-Cre*, and *Villin-Cre* (Jackson laboratory) mice were used to create tissue-specific conditional gankyrin knockout mice, designated here as *Mx1-Cre;Gankyrin^f/f^* and *Vil-Cre;Gankyrin^f/f^* mice. Induction of *Mx1-Cre* was achieved by 3 intraperitoneal injections of 300 μg of poly(I:C) (Sigma-Aldrich, St. Louis, MO) every other day to 4–8-week-old mice.

### Mouse treatment

*Mx1-Cre;Gankyrin^f/f^* mice and *Gankyrin^f/f^* mice were treated with poly(IC) as described previously [43]. Sex- and age-matched poly(IC)-treated *Mx1-Cre;Gankyrin^f/f^* mice (referred to as *Mx1-Cre;Gankyrin^f/f^* mice), poly(IC)-treated *Gankyrin^f/f^* mice, *Vil-Cre;Gankyrin^f/f^* mice and *Gankyrin^f/f^* mice (8–16 weeks old) received 2.5% (w/v) dextran sodium sulfate (DSS; molecular weight, 36,000–50,000 kDa; MP Biomedicals, Solon, OH) in drinking water for 7 days. Inflammatory cell infiltration score was assessed as described previously [6]. Isolation of lamina propria (LP) cells was performed as described previously [6]. As the protocol for the murine CAC model [6], mice were intraperitoneally (i.p.) injected with 12.5 mg/kg azoxymethane (AOM; Sigma-Aldrich). After 5 days, 2.0% DSS was included in the drinking water for 5 days, followed by 16 days of regular water. This cycle was repeated three times. Then, 1.5% DSS was included in the drinking water for 4 days, followed by 7 days of regular water. Upon sacrifice, the colon was excised from the ileocecal junction to the anus, cut open longitudinally, and prepared for histological evaluation. Colons were assessed macroscopically for polyps under a dissecting microscope. All animal procedures were performed according to approved protocols and in accordance with the recommendations for the proper care and use of laboratory animals. The animal study protocol was approved by the Medical Ethics Committee of Kindai University Faculty of Medicine and Institutional Animal Care and Use Committee of RIKEN Kobe.

### Cell culture

THP-1 cells, an immortalized line of human monocyte, were maintained in RPMI-1640 medium (Gibco, Carlsbad, CA) supplemented with 10% fetal bovine serum, containing penicillin (100 U/ml) and streptomycin (100 mg/ml) and transfection of gankyrin siRNA and SHP-1 siRNA (Santa Cruz, Dallas, TX) was carried out using X-treme GENE siRNA Transfection Reagent (Roche, Basel, Switzerland). Caco2 and U-2 OS cells were maintained in DMEM medium (Gibco) supplemented with 10% fetal bovine serum, containing penicillin (100 U/ml) and streptomycin (100 mg/ml) and transfected with plasmid using Fugene (Promega, Madison, WI) and X-treme GENE HP DNA transfection reagent (Roche). Cell viability was assessed using Cell Counting Kit-8 (Dojindo, Tokyo, Japan). Jurkat cells were transfected with a plasmid containing 3HA-gankyrin cDNA.

### Biochemical and immunochemical analyses

Real-time qPCR, immunoblotting, immunoprecipitation and immunohistochemistry were previously described [6, 44]. List of primer sequences and FACS protocols are mentioned in Supplementary Materials and Methods. The following antibodies were used: anti-actin, anti-FLAG, anti-biotin conjugated FLAG (Sigma-Aldrich); anti-Bmi1, anti-phospho-STAT3, anti-STAT3, anti-phospho- extracellular signal-regulated protein kinase (ERK), anti-ERK, anti-phospho-c-Jun N-terminal kinase (JNK), anti-JNK, anti-phospho-p38, anti-p38 (Cell Signaling, Danvers, MA); anti-HA, anti-biotin conjugated HA (Roche), anti-SHP-1 (R&D systems, Minneapolis, MN) and anti-CD3, anti-CD11b, anti-CD20, anti-CD68, anti-CD138 (BD bioscience, San Jose, CA). Generation of anti-gankyrin polyclonal antibody was previously described [45]. Immunohistochemistry was performed using ImmPRESS™ reagents (Vector Laboratory, Burlingame, CA) according to the manufacturer's recommendations.

Duolink fluorescence method was employed as per manufacturer's recommendations (Sigma Aldrich). Human gankyrin antibody and SHP-1 antibody (R&D systems) were used to assess gankyrin/SHP-1 interactions under confocal laser microscope.

Using the full length of gankyrin as bait, a cDNA library from human fetal intestine was screened by the yeast two-hybrid method, which was done by Invitrogen (Carlsbad, CA). Thirty-seven clones of yeast clones transformed with a human intestinal cDNA library were identified. Each clone proliferated on media containing the histidine inhibitor 3AT and was positive for β-galactosidase staining. DNA sequencing of the rescued plasmids revealed that two clones encoded SHP-1.

Immunofluorescent TUNEL staining was performed to measure apoptosis in paraffin-embedded sections using the *In Situ* Apoptosis Detection Kit as described by the manufacturer (Takara, Tokyo, Japan) and previous report [6]. Nuclei were stained with 4′, 6diamidino-2-phenylindole (DAPI) to count the total cells per crypt.

### Statistical analysis

Differences were analyzed using Student's *t*-test. To compare variables of more than 2 conditions, analysis of variance (ANOVA) with post-hoc Tukey-Kramer honestly significant difference (HSD) multiple comparison was applied. The relationship between the expression of several genes was analyzed by Spearman's rank correlation test. *P* values <0.05 were considered significant.

## SUPPLEMENTARY MATERIALS FIGURES AND TABLES



## Abbreviations

Ascl2: achaete-scute complex homolog 2
AOM: azoxymethane
Bmi1: B cell-specific Moloney murine leukemia virus insertion site 1
CAC: colitis-associated cancer
CD: Crohn's disease
DSS: dextran sodium sulfate
ERK: extracellular signal-regulated protein kinase
IBD: inflammatory bowel disease
JNK: c-Jun N-terminal kinase
Lgr5: leucine-rich repeat-containing G-protein-coupled receptor 5
LP: lamina propria
Pim1: provirus integration site for Moloney murine leukemia virus 1
Sox9: sex-determining region Y (SRY)-box 9
STAT3: signal transducer and activator of transcription-3
UC: ulcerative colitis
