# Supplementary material for: The oncoprotein gankyrin promotes the development of colitis-associated cancer through activation of STAT3
Source: Oncotarget. 2017 Feb 1;8(15):24762–76. doi: 10.18632/oncotarget.14983 (PMC5421886; doi:10.18632/oncotarget.14983)
Supplement: Supplementary file 1 [file oncotarget-08-24762-s001.pdf]

The oncoprotein gankyrin promotes the development of colitis-associated cancer through activation of STAT3

SUPPLEMENTARY DATA

SUPPLEMENTARY MATERIALS AND METHODS

Primers for PCR

RT-PCR was performed with the following sets of primers: for human β-actin, 5'-tccctggagaagagctacga-3' and 5'-aggaaggaaggctggaagag-3'; for human TNF-α, 5'-aacctcct ctctgccatcaa-3' and 5'-ggaagacccctcccagatag-3'; for human Lgr5, 5'-gtcccacttcctgcatgtct-3' and 5'-ctcaggctcaccagatcc tc-3'; for human Bmi1, 5'-ccagggcttttcaaaaatga-3' and 5'-attagagccattggcagcat-3'; for human IL-6, 5'-aaagag gcactggcagaaaa-3' and 5'-tttcaccaggcaagtctcct-3'; for human IL-17, 5'-catgaactctgtccccatcc-3' and 5'-cccacggacaccagta tctt-3'; for human IL-23, 5'-tctccttctccgcttcaaaa-3' and 5'-ttagggactcagggttgctg-3'; for human gankyrin, 5'-aaaactgc tggtgtcccaag-3' and 5'-attaaacccaggccacctttt-3'; for human Pim1, 5'-caggcagagggtctcttcag-3' and 5'-tggatttcttcgaag gttgg-3'; for mouse β-actin, 5'-gaccctgaagtaccccattgaa-3' and 5'-aaggtgtggtgccagatcttct-3'; for mouse TNF-α, 5'-gaccaggctgtcgctacatca-3' and 5'-cgtaggcgattacagtcac gg-3'; for mouse IL-6, 5'-gaggataccactcccaacagacc-3' and 5'-aagtgcatcatcgttgttcataca-3'; for mouse IL-17, 5'-agctggaccaccatgaat-3' and 5'-agcatcttctcgaccctgaa-3'; for mouse IL-23, 5'-gacccacaaggactcaagga-3' and 5'-aggctcccc tttgaagatgt-3'; for mouse Pim1, 5'-gccctcctttgaagaaatcc-3' and 5'-cggtgacagactgtgcagat-3'; for mouse cMyc, 5'-ccag atccctgaattggaaa-3' and 5'-tcgtctgcttgaatggacag-3'; for mouse Sox9, 5'-ctgaagaaggagagcgagga-3' and 5'-gtcca gtcgtagcccttcag-3'; for mouse Ascl2, 5'-tggtaaacttgggct tccag-3' and 5'-gcagcgtctccaccttactc-3'; and for mouse gankyrin, 5'-aaagatgacgcaggttggtc-3' and 5'-tgtgcacctttcac cagaag-3'.

PCR for genotyping was performed with the following primers: 5'-acgactcctggtccaactgtaacc-3' and 5'-atctccatcgcgctgtctcttaac-3'. PCR fragments of wild-type allele and floxed allele was 142 kb and 200 kb, respectively.

FACS analysis

Preparation of the single cell suspension from spleen was performed as follows; spleen was put on the cell strainer and ACK lysing buffer (Thermo Fisher Scientific, Waltham, MA) was added to remove red blood cells. Spleen was tamped with the rubber end of a plunger from a 2.5 ml syringe against the cell strainer to make single splenocytes. ACK lysing buffer was added and the cell suspension was left for 3 min at room temperature, washed and centrifuged. Cell number was counted by hematocytometer and cells (10^8 cells/ml) are ready for staining.

Isolated splenocytes were stained with FITC or PE-conjugated CD20 Ab (eBioscience, San Diego, CA), CD3 Ab (eBioscience), CD11b Ab (BD Biosciences, San Jose, CA), rat IgG (BioLegend, San Diego, CA), or hamster IgG (BioLegend). Flow cytometric analysis was performed using an Accuri C6 flow cytometer (BD Biosciences) and CFlow Plus software (BD Biosciences). Fixation and Permialization solution was used to stain for intracellular protein gankyrin as described by the manufacturer (BD Biosciences).

SUPPLEMENTARY FIGURES AND TABLE

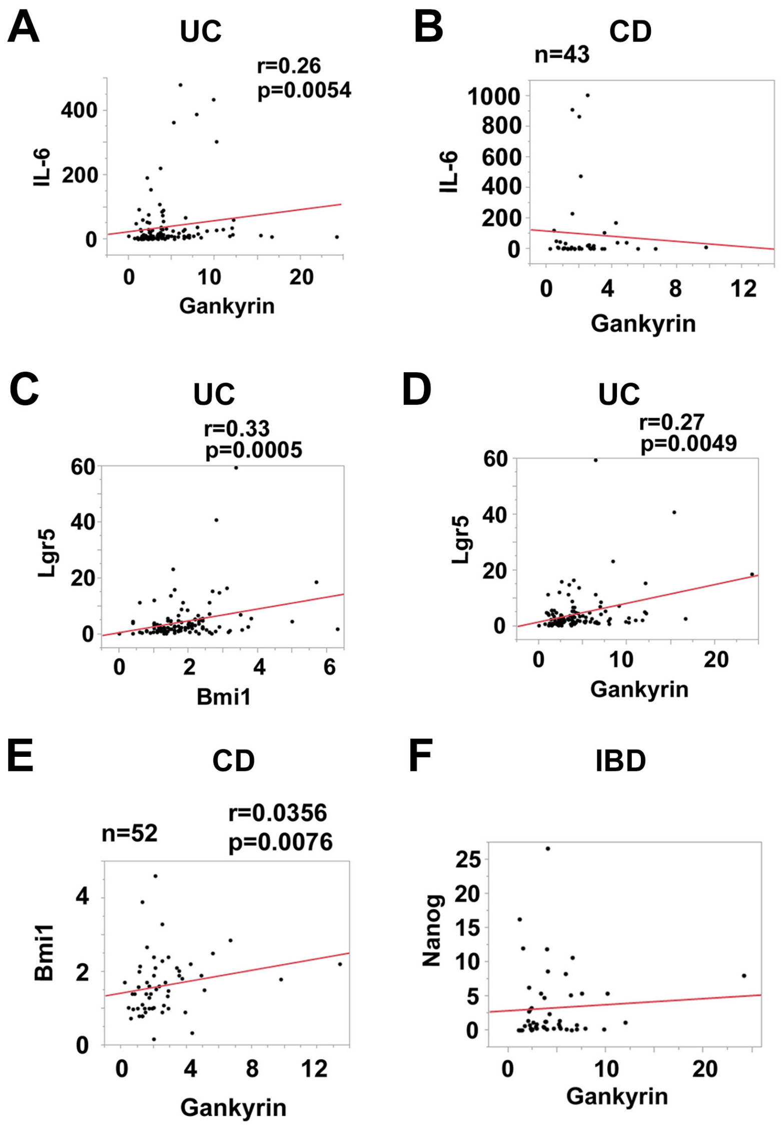

Supplementary Figure 1: Scatter plot of relative mRNA levels of indicated genes in the colonic mucosa of patients with ulcerative colitis (UC), Crohn's disease (CD) or inflammatory bowel disease (IBD).

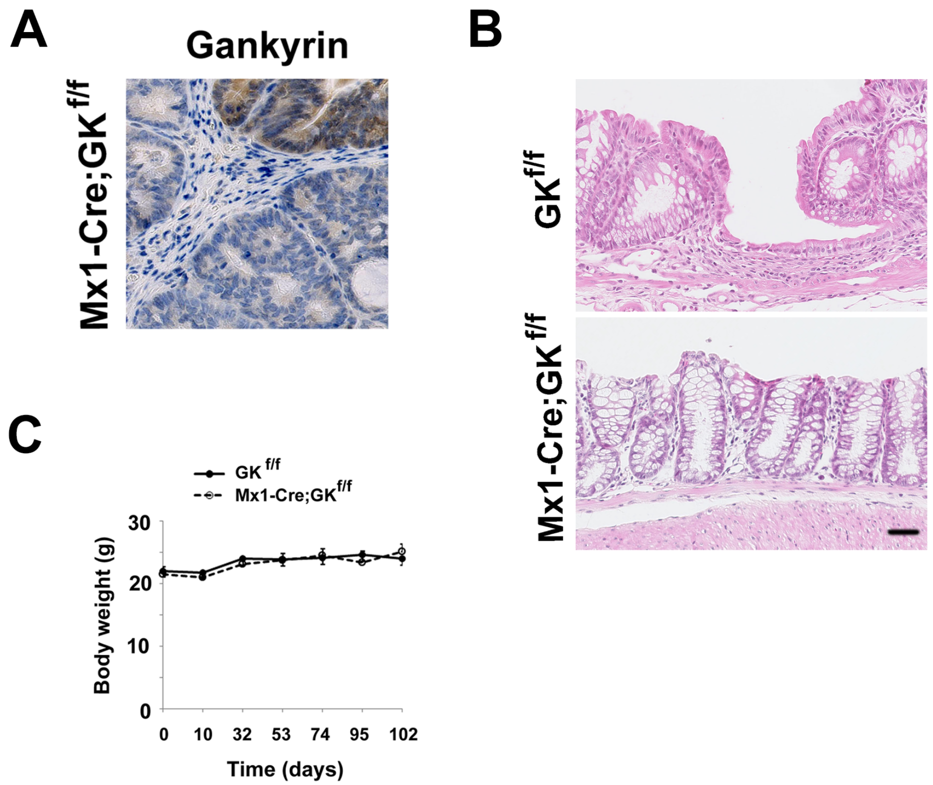

Supplementary Figure 2: *Mx1-Cre;Gankyrin$^{f/f}$* (Mx1-Cre;GK$^{f/f}$) mice and *Gankyrin$^{f/f}$* (GK$^{f/f}$) mice were challenged with AOM and DSS. A. Images of immunohistochemical detection of gankyrin in tumors from AOM/DSS-treated *Mx1-Cre;Gankyrin$^{f/f}$* (Mx1-Cre;GK$^{f/f}$) mice. **B.** Representative photographs of H&E-stained non-tumor colons of *Mx1-Cre;Gankyrin$^{f/f}$* (Mx1-Cre;GK$^{f/f}$) mice and *Gankyrin$^{f/f}$* (GK$^{f/f}$) mice. **C.** Body weight of *Mx1-Cre;Gankyrin$^{f/f}$* (Mx1-Cre;GK$^{f/f}$) mice and *Gankyrin$^{f/f}$* (GK$^{f/f}$) mice. Data are means ± SEM.

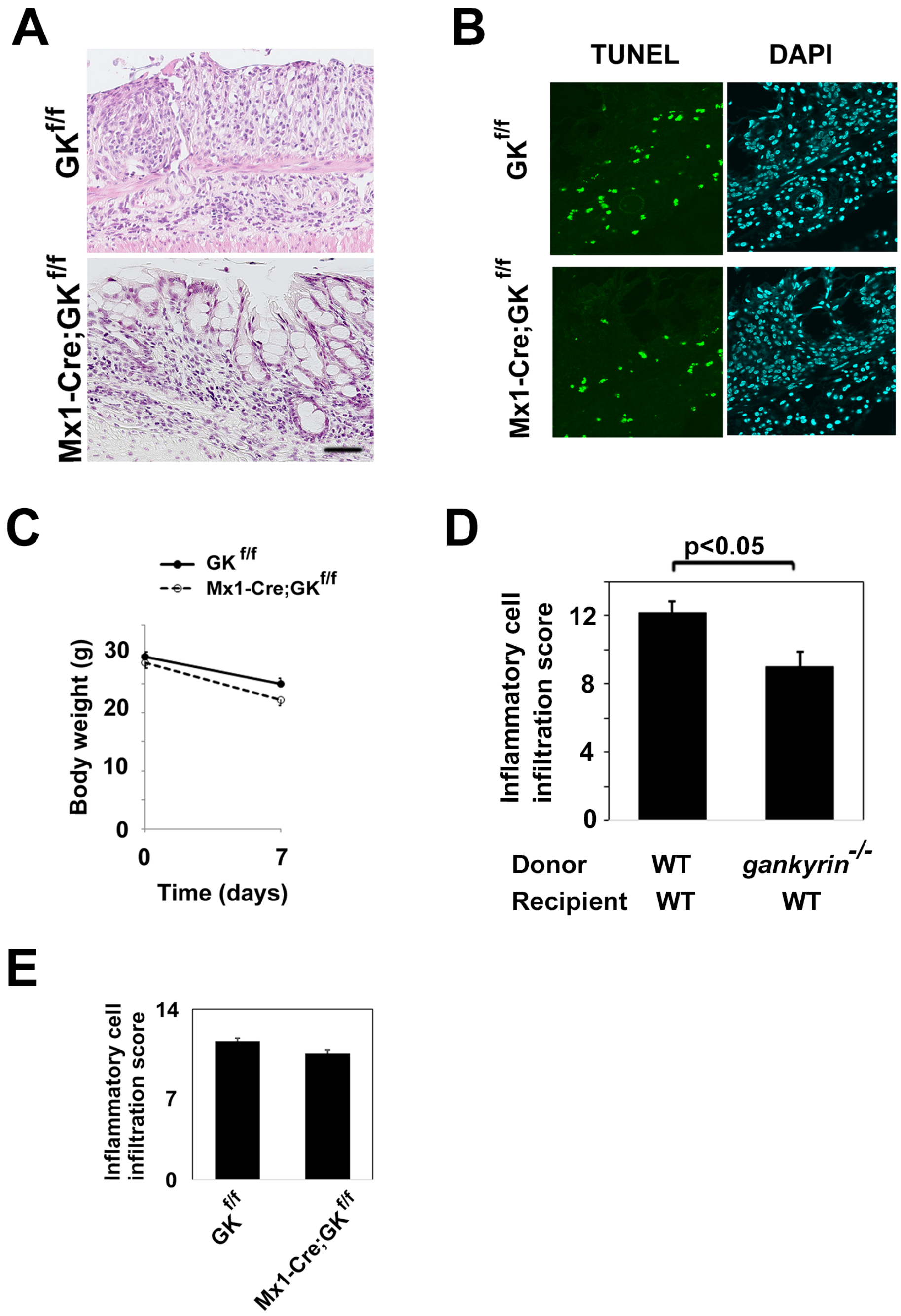

Supplementary Figure 3: *Mx1-Cre;Gankyrin*$^{f/f}$ (Mx1-Cre;GK$^{f/f}$) mice and *Gankyrin*$^{f/f}$ (GK$^{f/f}$) mice were challenged with DSS for 7 days. A, B. Representative HE (A) and TUNEL staining (B) images of colonic tissues from DSS-treated *Mx1-Cre;Gankyrin*$^{f/f}$ (Mx1-Cre;GK$^{f/f}$) mice and *Gankyrin*$^{f/f}$ (GK$^{f/f}$) mice. **C.** Body weight was measured. Data are means ± SEM. **D.** *Gankyrin*$^{f/f}$ (WT) mice with wild type (WT) or gankyrin-deficient (*gankyrin*$^{-/-}$) bone marrow were generated by bone marrow transplantation. Inflammatory cell infiltration score was assessed as described previously [6]. Data are means ± SEM. **E.** To discuss the clinical implication of gankyrin inhibition, we injected poly(I:C) into *Mx1-Cre;Gankyrin*$^{f/f}$ mice and deleted gankyrin after the initiation of DSS treatment. Inflammatory cell infiltration score was measured in *Mx1-Cre;Gankyrin*$^{f/f}$ (Mx1-Cre;GK$^{f/f}$) mice and *Gankyrin*$^{f/f}$ (GK$^{f/f}$) mice. Data are means ± SEM.

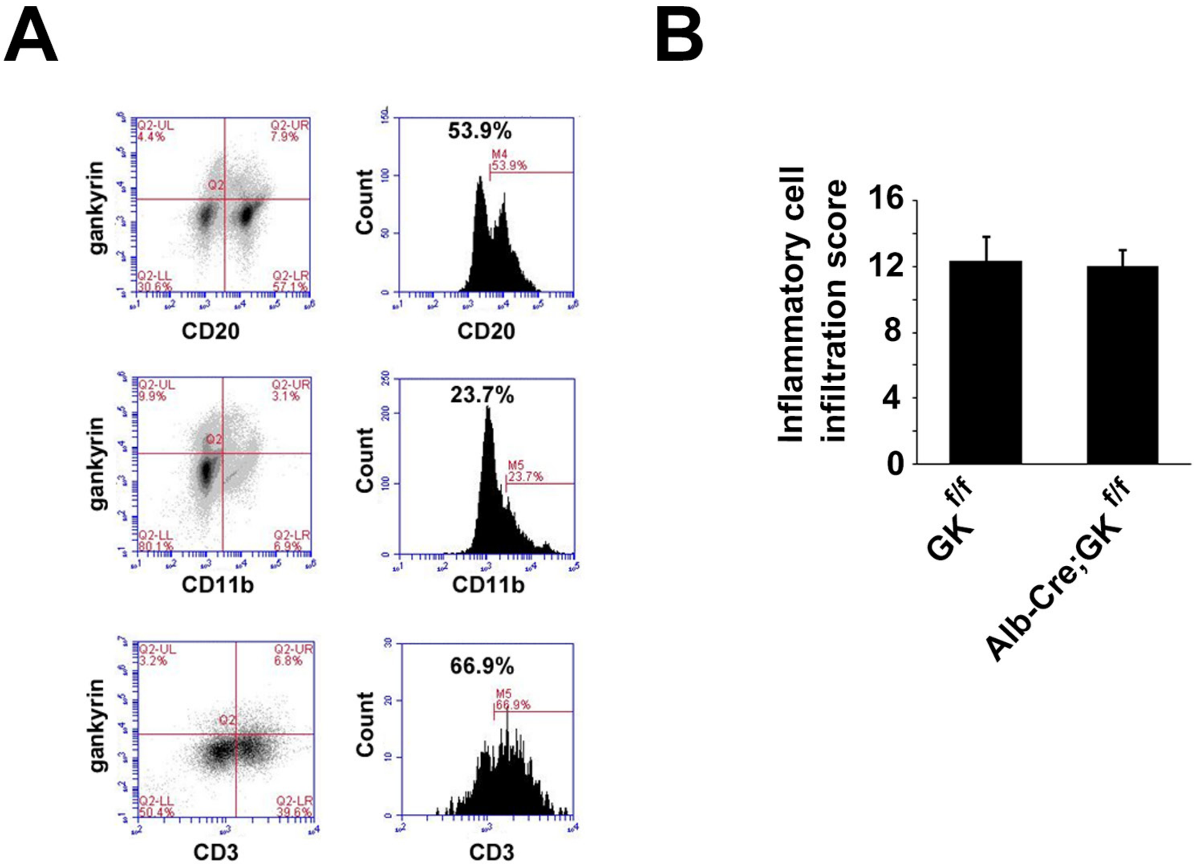

Supplementary Figure 4: A. Isolated splenocytes were stained with FITC or PE-conjugated CD20 Ab (eBioscience, San Diego, CA), CD3 Ab (eBioscience), CD11b Ab (BD Biosciences, San Jose, CA), rat IgG (BioLegend, San Diego, CA), or hamster IgG (BioLegend). Flow cytometric analysis was performed using an Accuri C6 flow cytometer (BD Biosciences) and CFlow Plus software (BD Biosciences). **B.** *Alb-Cre;Gankyrin*$^{f/f}$ (Alb-Cre;GK$^{f/f}$) mice and *Gankyrin*$^{f/f}$ (GK$^{f/f}$) mice were challenged with DSS for 7 days and inflammatory cell infiltration score was measured. Data are means ± SEM.

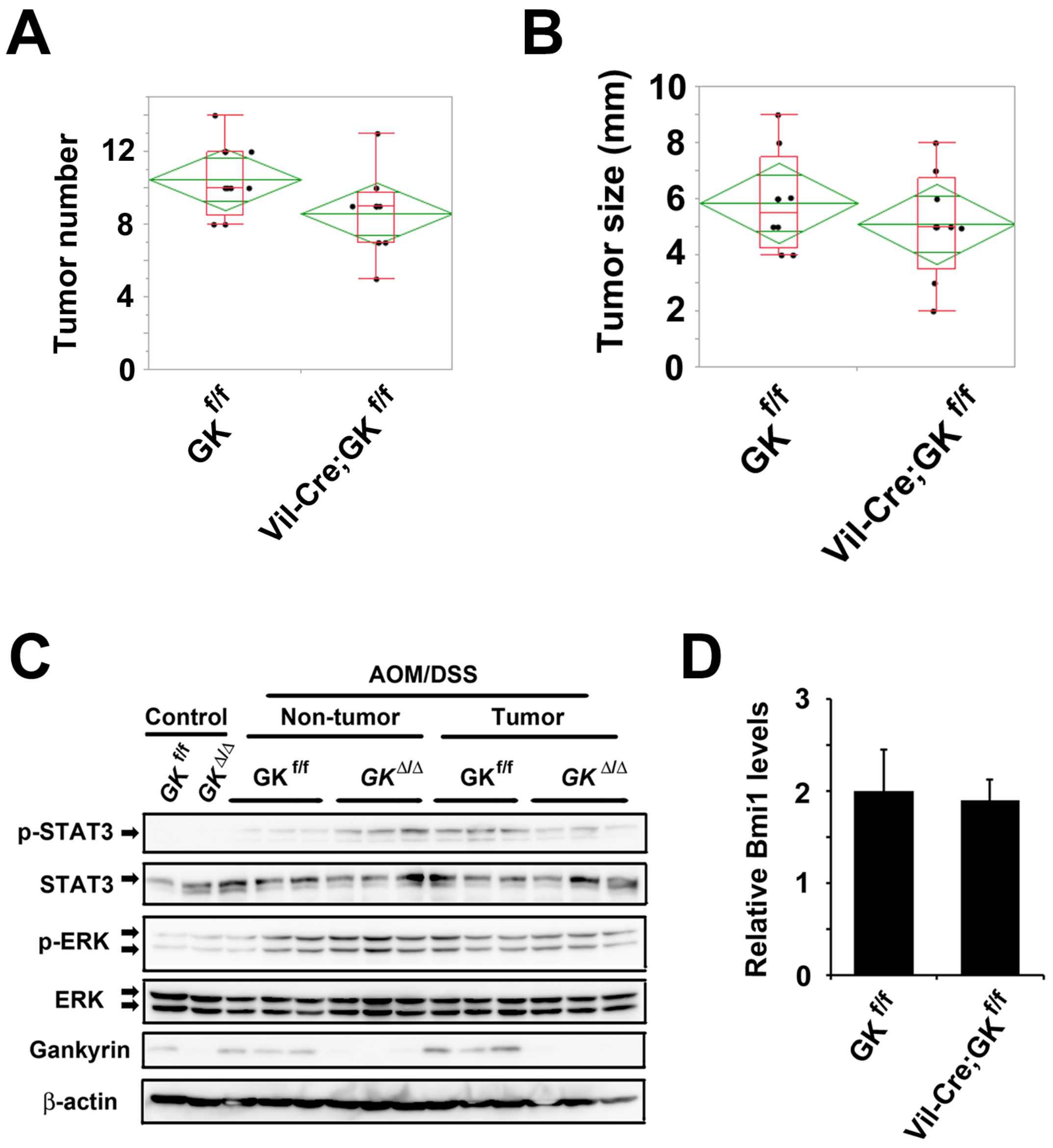

Supplementary Figure 5: *Villin-Cre;Gankyrin$^{f/f}$* (Vil-Cre;GK$^{f/f}$, GK$^{\Delta/\Delta}$) mice and *Gankyrin$^{f/f}$* (GK$^{f/f}$) mice were challenged with AOM and DSS. Tumor number **A.** and maximum size (**B.**, GK$^{f/f}$ mice, n = 8; Vil-Cre;GK$^{f/f}$ mice, n = 8). **C.** Homogenates of non-treated colons (Control), non-tumor colon tissues (Non-tumor) and tumors (Tumor) were gel-separated and immunoblotted with the indicated antibodies. **D.** Relative amounts of Bmi1 mRNA in non-tumor colons were determined by real-time qPCR and normalized to the amount of actin mRNA. The amount of mRNA in the untreated colon was given an arbitrary value of 1.0. Data are means ± SEM.

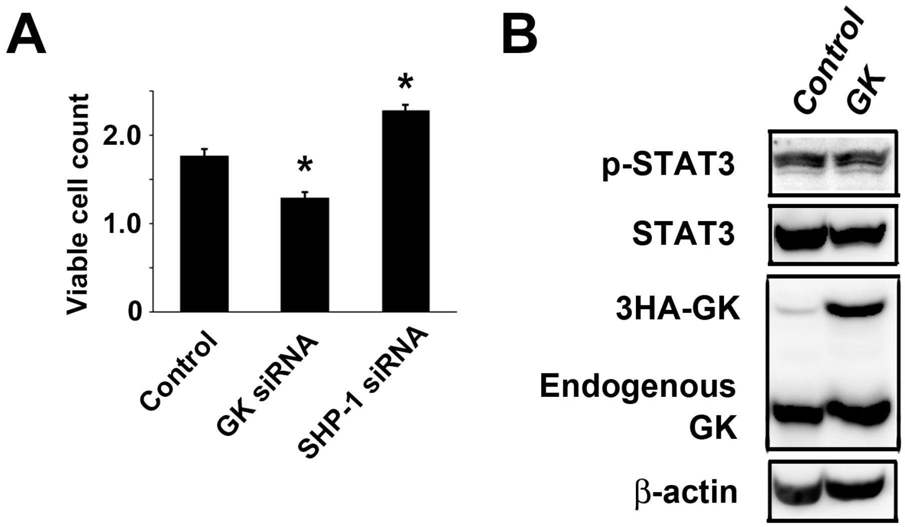

Supplementary Figure 6: A. Colorectal cancer Caco2 cells were transfected with a plasmid containing gankyrin siRNA (GK siRNA) and SHP-1 siRNA. Twenty four hours later, viable cells were counted. Data are means ± SEM. **B.** Jurkat cells, an immortalized line of human T lymphocyte, were transfected with a plasmid containing 3HA-gankyrin cDNA and cells were harvested 48 hours later. Homogenates of cells were gel-separated and immunoblotted with the indicated antibodies.

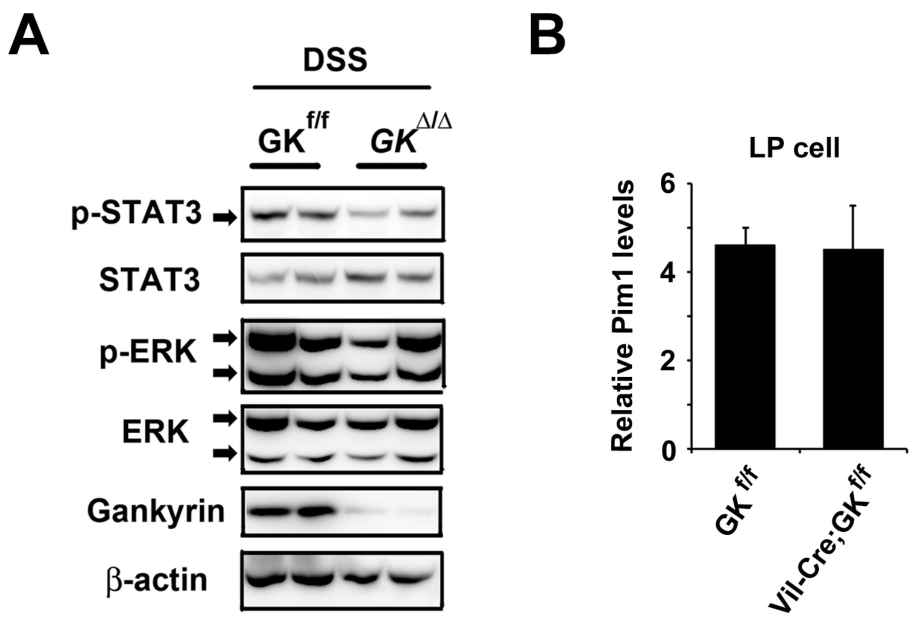

Supplementary Figure 7: ***Villin-Cre;Gankyrin$^{f/f}$*** **(Vil-Cre;GK$^{f/f}$, GK$^{\Delta/\Delta}$) mice and** ***Gankyrin$^{f/f}$*** **(GK$^{f/f}$) mice were challenged with DSS for 7 days.** **A.** Homogenates of colons were gel-separated and immunoblotted with the indicated antibodies. **B.** lamina propria (LP) cells were isolated from DSS-treated *Villin-Cre;Gankyrin$^{f/f}$* (Vil-Cre;GK$^{f/f}$) mice and *Gankyrin$^{f/f}$* (GK$^{f/f}$) mice and Pim1 mRNA expression was analyzed by real-time qPCR. The mRNA expression levels in LP cells from non-treated WT mice were set as 1.

Supplementary Table 1: Baseline characteristics of the patients

	Control (n=54)	In Remission (n=47)	Non-refractory (n=115)	Refractory (n=128)
Age (years)	57 ± 11	44 ± 16	41 ± 9	44 ± 17
Sex (M/F)	31/23	22/25	69/46	71/57
Disease duration (years)		7.8 ± 6.5	4.6 ± 6.7	7.2 ± 7.9
Extent of disease				
UC-pancolitis		25	64	63
UC-left colitis		10	27	31
UC-proctitis		7	15	11
CD-ileitis		2	5	6
CD-ileocolitis		2	3	14
CD-colitis		1	1	3
CRP (mg/dl)	0.31 ± 0.59	0.53 ± 1.6	0.95 ± 1.6	1.1 ± 1.9
Albumin (g/dl)	4.1 ± 0.40	4.2 ± 0.55	3.7 ± 0.72*	3.7 ± 0.64*
Mayo endoscopic score		0.3 ± 0.46	2.3 ± 0.58*	2.4 ± 0.56*
Previous or concomitant medication use				
5-ASA		47	115	128
Steroid		10	40	43
Anti-TNF		11	21	59
IM		10	28	30

Some of results were expressed as means ± SD. Ulcerative colitis; UC. Crohn's disease; CD. C-reactive protein; CRP. Immunomodulator; IM. *$P < 0.05$ compared with IBD patients in remission.
